# Changes in characteristics and outcomes of critically ill COVID-19 patients in Tyrol (Austria) over 1 year

**DOI:** 10.1007/s00508-021-01945-5

**Published:** 2021-10-18

**Authors:** Timo Mayerhöfer, Sebastian J. Klein, Andreas Peer, Fabian Perschinka, Georg F. Lehner, Julia Hasslacher, Romuald Bellmann, Lukas Gasteiger, Markus Mittermayr, Stephan Eschertzhuber, Simon Mathis, Anna Fiala, Dietmar Fries, Armin Kalenka, Eva Foidl, Walter Hasibeder, Raimund Helbok, Lukas Kirchmair, Birgit Stögermüller, Christoph Krismer, Tatjana Heiner, Eugen Ladner, Claudius Thomé, Christian Preuß-Hernandez, Andreas Mayr, Agnes Pechlaner, Miriam Potocnik, Bruno Reitter, Jürgen Brunner, Stefanie Zagitzer-Hofer, Alexandra Ribitsch, Michael Joannidis

**Affiliations:** 1 Division of Intensive Care and Emergency Medicine, Department of Internal Medicine, Medical University Innsbruck, Anichstr. 35, 6020 Innsbruck, Austria; 2 Doctoral College Medical Law and Healthcare, Faculty of Law, University Innsbruck, Innsbruck, Austria; 3 Department of Anesthesia and Critical Care Medicine, Medical University Innsbruck, Innsbruck, Austria; 4 Department of Anesthesia and Intensive Care Medicine, Hospital Hall, Hall, Austria; 5 Department of General and Surgical Intensive Care Medicine, Medical University Innsbruck, Innsbruck, Austria; 6 Department of Anesthesia and Intensive Care Medicine, Hospital Kufstein, Kufstein, Austria; 7 Department of Anesthesiology and Critical Care Medicine, Hospital St. Vinzenz Zams, Zams, Austria; 8 Department of Neurology, Medical University Innsbruck, Innsbruck, Austria; 9 Department of Anesthesia and Critical Care Medicine, Hospital Schwaz, Schwaz, Austria; 10 Department of Internal Medicine, Hospital St. Vinzenz Zams, Zams, Austria; 11 Department of Anesthesia and Intensive Care Medicine, Hospital Reutte, Reutte, Austria; 12 Department of Anesthesia and Intensive Care Medicine, Hospital Lienz, Lienz, Austria; 13 Department of Neurosurgery, Medical University Innsbruck, Innsbruck, Austria; 14 Department of Anesthesia and Intensive Care Medicine, Hospital St. Johann in Tyrol, St. Johann in Tyrol, Austria; 15 Department of Pediatrics, Medical University Innsbruck, Innsbruck, Austria; 16 Department of Internal Medicine, Hospital Hall, Hall, Austria; 17 Department of Internal Medicine, Hospital Lienz, Lienz, Austria

**Keywords:** Elderly, SARS-CoV‑2, Mechanical ventilation, Acute kidney injury, Second wave

## Abstract

**Background:**

Widely varying mortality rates of critically ill Coronavirus disease 19 (COVID-19) patients in the world highlighted the need for local surveillance of baseline characteristics, treatment strategies and outcome. We compared two periods of the COVID-19 pandemic to identify important differences in characteristics and therapeutic measures and their influence on the outcome of critically ill COVID-19 patients.

**Methods:**

This multicenter prospective register study included all patients with a SARS-CoV‑2 infection confirmed by polymerase chain reaction, who were treated in 1 of the 12 intensive care units (ICU) from 8 hospitals in Tyrol, Austria during 2 defined periods (1 February 2020 until 17 July: first wave and 18 July 2020 until 22 February 2021: second wave) of the COVID-19 pandemic.

**Results:**

Overall, 508 patients were analyzed. The majority (*n* = 401) presented during the second wave, where the median age was significantly higher (64 years, IQR 54–74 years vs. 72 years, IQR 62–78 years, *p* < 0.001). Invasive mechanical ventilation was less frequent during the second period (50.5% vs 67.3%, *p* = 0.003), as was the use of vasopressors (50.3% vs. 69.2%, *p* = 0.001) and renal replacement therapy (12.0% vs. 19.6%, *p* = 0.061), which resulted in shorter ICU length of stay (10 days, IQR 5–18 days vs. 18 days, IQR 5–31 days, *p* < 0.001). Nonetheless, ICU mortality did not change (28.9% vs. 21.5%, *p* = 0.159) and hospital mortality even increased (22.4% vs. 33.4%, *p* = 0.039) in the second period. Age, frailty and the number of comorbidities were significant predictors of hospital mortality in a multivariate logistic regression analysis of the overall cohort.

**Conclusion:**

Advanced treatment strategies and learning effects over time resulted in reduced rates of mechanical ventilation and vasopressor use in the second wave associated with shorter ICU length of stay. Despite these improvements, age appears to be a dominant factor for hospital mortality in critically ill COVID-19 patients.

**Supplementary Information:**

The online version of this article (10.1007/s00508-021-01945-5) contains supplementary material, which is available to authorized users.

## Introduction

As of May 2021, more than 600,000 patients tested positive and around 10,000 deaths are attributed to Coronavirus disease 19 (COVID-19) in Austria [1]. While in the beginning of the pandemic the number of SARS-CoV‑2 positive patients requiring intensive care was unknown, the rate settled at around 1–2% during the second period [2].

Due to numerous influencing factors different cohorts from various areas have demonstrated widely varying mortality rates and characteristics of intensive care unit (ICU) patients. A meta-analysis published in June 2020 found ICU mortality rates ranging from 14–84% [3], which has shown the importance of observing regional conditions separately in order to have a better understanding of major factors influencing outcome [4].

Since the beginning of the COVID-19 pandemic, countless studies about therapeutic strategies and the management of ICU patients have been initiated and published [5, 6]. With the RECOVERY trial corticosteroid treatment has shown a mortality benefit in patients requiring respiratory support and has changed therapeutic strategies for critically ill COVID-19 patients. Guidelines have been continuously updated, adapting recommendations based on the best available evidence.

In Tyrol, Austria, comprising 750,000 inhabitants we established the Tyrolean COVID-19 intensive care registry (Tyrol-CoV-ICU-Reg) at the beginning of the first surge of the pandemic in March 2020. Characteristics and outcomes of the first period have previously been published [4]. Since then, we continued the registry throughout the second period of the COVID-19 crisis.

We assumed that the rapid growth in knowledge as well as the dynamic of the ongoing pandemic may result in different and changing patient characteristics and outcomes of critically ill patients over time. Therefore, the aim of this study was to evaluate critically ill patients with COVID-19, treated in any of the 12 ICUs dedicated to COVID-19 in Tyrol, Austria and to identify important changes between the two waves in baseline characteristics, treatment strategies and outcomes.

## Methods

### Study design and participants

The Tyrol-CoV-ICU-Reg is a multicenter prospective register study and collects data from 13 ICUs (8 hospitals, list of all ICUs in the electronic supplementary material, ESM) providing critical care for COVID-19 patients in Tyrol. Methods and study design have been published previously [4].

Patients with a SARS-CoV‑2 infection confirmed by polymerase chain reaction (PCR) and admitted to an ICU or intermediate care unit in Tyrol (Austria) from 1 February 2020 until 22 February 2021 were included in this analysis. Patients under 18 years of age have been excluded from the main analysis and are reported separately in the ESM (Tables S1–3).

A total of 13 ICUs (from 8 hospitals in the region of Tyrol, Austria) including the University Hospital of Innsbruck with up to 5 parallel ICUs provided data for the Tyrol-CoV-ICU-Reg. For this study, multiple hospitalizations of patients in different ICUs were combined.

This registry was approved by the local ethics committee (Nr. 1099/2020).

### Data collection and definitions

**Fig. 1:**
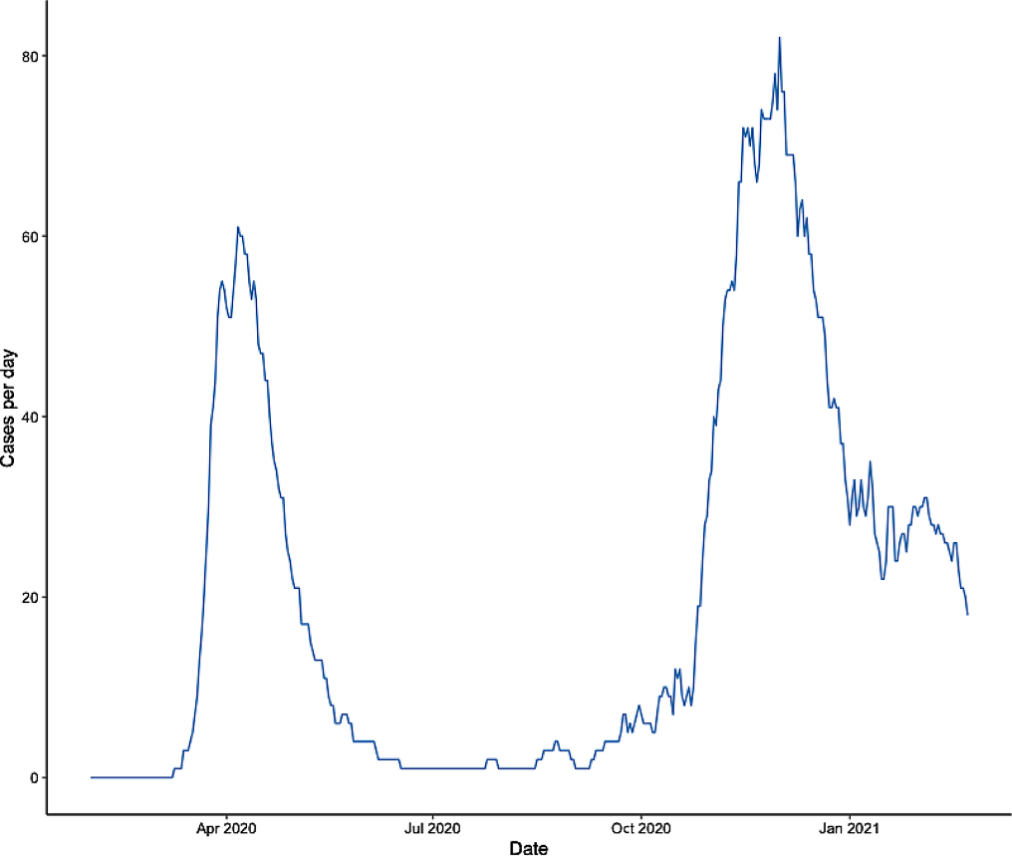
Active cases of critically ill COVID-19 patients per day in Tyrol, Austria, over 1 year

For comparison of the different periods of the pandemic two waves were defined for the analysis. According to the rise and fall of active cases of ICU patients in Tyrol (Austria) during the past year (Fig. 1), the two periods were set from 1 February 2020 until 17 July 2020 (first wave) and from 18 July 2020 until 22 February 2021 (second wave). The cohort of the first wave has already been reported in detail [4] and is described again in this manuscript for reasons of comparison.

Data for the second period of this registry were collected via electronic case report forms using REDCap electronic data capture tools hosted at the Department for Medical Statistics, Informatics and Health Economics, Medical University Innsbruck [7, 8].

The collected baseline characteristics were age, sex, weight, height, glycated hemoglobin (HbA1c), comorbidities, smoking habits, date of symptom onset, location before admission, date of hospital and ICU admission, Simplified Acute Physiology Score (SAPS) III and Sequential Organ Failure Assessment (SOFA) score at admission.

During the ICU stay, COVID-19 typical radiological alterations (x-ray or computed tomography), respiratory support and therapies (high flow nasal cannula, HFNC, noninvasive ventilation, NIV, mechanical ventilation, prone positioning and use of neuromuscular blockade), acute kidney injury (AKI) as well as renal replacement therapy (RRT), extracorporeal membrane oxygenation (ECMO), corticosteroids and changes in the therapy goal were recorded.

Patients were followed until death or hospital discharge, whichever occurred earlier.

The AKI was defined and staged according to the Kidney Disease: Improving Global Outcome (KDIGO) criteria [9].

### Statistical Analysis

Statistical analysis was conducted using R software (version 3.4.0, R Foundation for Statistical Computing, Vienna, Austria). Categorical variables are presented as numbers and corresponding percentages. Continuous data are expressed as median with interquartile range (IQR). Shapiro-Wilk test was used to test for normal distribution (ND). Continuous and categorical variables were compared using Student’s t-test for normally distributed and Mann-Whitney U‑test or χ2-test for not normally distributed data.

We evaluated possible clinically relevant predictors for hospital mortality with univariate and multivariate logistic regression. Variables with a *p* value < 0.05 in univariate analysis were entered in a multivariate model. Age, the period of presentation (first or second wave), frailty, time from symptom onset to ICU admission and the number of comorbidities were included in the multivariate model. Adjusted odds ratios (OR) and confidence intervals (CI) were calculated.

Hospital mortality was further analyzed using Kaplan-Meier survival analysis. Patients were divided into two groups according to the median age (older and younger) and were analyzed in univariate analysis. Differences were assessed by the log-rank test.

Additionally, we performed 1:1 propensity score matching to estimate the effect of the period of presentation (first or second wave) on hospital mortality accounting for multiple baseline characteristics (Supplemental Table 11). For this purpose, we excluded patients with missing values in certain variables (SAPS III score, frailty and patients from nursing home), as this affected only very few cases. For missing values in the time from symptom onset to ICU admission and in BMI we used mean imputation. Supplemental Table 11 lists the variables included in the propensity score matching analysis and their corresponding standardized differences before and after matching. The effect of the period of presentation (first or second wave) on hospital mortality was evaluated with conditional logistic regression analysis (Supplemental Table 12).

For all tests, a two-tailed *p* value < 0.05 was considered statistically significant.

## Results

### Patient characteristics and study population

Baseline characteristics are presented in Table 1. In total 512 critically ill patients were admitted to 1 of the 13 study ICUs (5 ICUs at central university hospital, 7 ICUs from regional hospitals and 1 pediatric ICU at the central university hospital) from 9 March 2020 until 22 February 2021. Four patients were not included in main analysis due to age < 18 years and are reported in the ESM Tables S1–3.

**Table 1:** Baseline characteristics of 508 critically ill COVID-19 patients stratified by two waves [4]

	Overall	First wave	Second wave	*P* value
*n*	508	107	401	–
*Sex: male/female (%)*	356/152 (70.1/29.9)	77/30 (72.0/28.0)	279/122 (69.6/30.4)	0.719
*Age, years median (IQR)*	71.00 (60.00–78.00)	64.00 (54.00–74.00)	72.00 (62.00–78.00)	<0.001
**Comorbidities**				
Hypertension (%)	327 (64.4)	71 (66.4)	256 (63.8)	0.712
Cardiovascular (%)	217 (42.7)	45 (42.1)	172 (42.9)	0.964
Diabetes (%)	–	–	–	0.066
– Prediabetes	15 (3.0)	2 (1.9)	13 (3.2)	–
– DM type I	5 (1.0)	1 (0.9)	4 (1.0)	–
– DM type II	114 (22.4)	16 (15.0)	98 (24.4)	–
– DM (other type)	1 (0.2)	1 (0.9)	0 (0.0)	–
Renal (%)	108 (21.3)	21 (19.6)	87 (21.7)	0.740
Neurological (%)	75 (14.8)	11 (10.3)	64 (16.0)	0.187
Liver (%)	38 (7.5)	7 (6.5)	31 (7.7)	0.835
Hematological malignancy (%)	26 (5.1)	3 (2.8)	23 (5.7)	0.329
Immunosuppression (%)	36 (7.1)	11 (10.3)	25 (6.2)	0.216
Nonhematological malignancy (%)	40 (7.9)	5 (4.7)	35 (8.7)	0.246
COPD (%)	70 (13.8)	14 (13.1)	56 (14.0)	0.939
Asthma (%)	21 (4.1)	7 (6.5)	14 (3.5)	0.256
Respiratory—other (%)	41 (8.1)	13 (12.1)	28 (7.0)	0.123
Overweight (BMI ≥25) (%)	363 (74.1)	76 (76.0)	287 (73.6)	0.717
Obesity (BMI ≥30) (%)	150 (30.6)	25 (25.0)	125 (32.1)	0.214
*No known comorbidity (%)*	47 (9.6)	14 (14.1)	33 (8.5)	0.128
*Number of comorbidities, n median (IQR)*	2.00 (1.00–4.00)	2.00 (1.00–3.00)	3.00 (1.00–4.00)	0.069
*BMI, kg/m*^*2*^* median (IQR)*	27.52 (24.98–30.86)	26.83 (25.08–30.15)	27.73 (24.97–31.04)	0.313
*HbA1c, % median (IQR)*	6.30 (5.90–6.80)	6.20 (5.70–6.70)	6.30 (5.90–7.00)	0.082
**Risk factors**				
Active smoking (%)	43 (9.9)	12 (12.8)	31 (9.1)	0.384
Previous smoking (%)	43 (9.9)	12 (12.8)	31 (9.1)	0.384
Patient from nursing home (%)	14 (2.8)	0 (0.0)	14 (3.5)	0.101
Frailty				0.363
– Fully independent daily living (%)	422 (84.4)	92 (86.0)	330 (84.0)	–
– Pre-frail (%)	70 (14.0)	12 (11.2)	58 (14.8)	–
– Frail (%)	8 (1.6)	3 (2.8)	5 (1.3)	–
*COVID-19 primary reason for hospital admission (%)*	422 (83.1)	91 (85.0)	331 (82.5)	0.640
*COVID-19 typical findings in chest x‑ray (%)*	458 (92.0)	98 (92.5)	360 (91.8)	0.995
*COVID-19 typical findings in computed tomography (%)*	256 (87.7)	52 (81.2)	204 (89.5)	0.120
*SOFA score, median (IQR)*	5.00 (4.00-8.00)	6.00 (4.00–10.00)	5.00 (4.00–8.00)	0.107
*SAPS III score, median (IQR)*	56.00 (49.00–64.00)	56.00 (49.00–64.00)	55.00 (49.00–64.00)	0.868
*Time from symptom onset to hospital admission, days median (IQR)*	6.00 (3.00–9.00)	6.50 (4.00–9.00)	6.00 (3.00–9.00)	0.308
*Time from symptom onset to ICU admission, days median (IQR)*	8.00 (5.00–11.00)	8.00 (5.00–11.00)	8.00 (5.00–11.00)	0.636

The 107 patients from the first wave have been previously reported [4].

*IQR* interquartile range, *DM* diabetes mellitus, *COPD* chronic obstructive pulmonary disease, *BMI* body mass index, *HbA1c* glycated hemoglobin, *SOFA* sequential organ failure assessment, *SAPS* simplified acute physiology score, *ICU* intensive care unit

The majority presented during the second period (*n* = 401/508). All patients had SARS-CoV‑2 infection confirmed by polymerase chain reaction (PCR). The overall median age was 71 years (IQR 60–78 years) and the majority of patients were male (*n* = 356, 70.1%). At admission median SAPS III score and median SOFA score were 56 (IQR 49–64) and 5 (IQR 4–8), respectively. COVID-19 was the primary reason for hospital admission in 83.1% (*n* = 422) of the patients, 92% (*n* = 458) showed typical radiological changes in the chest x‑ray and 87.7% (*n* = 256) in the computed tomography during the ICU stay. The median time from symptom onset to hospital and ICU admission was 6 days (IQR 3–9days) and 8 days (IQR 5–11 days), respectively.

The median body mass index (BMI) was 27.52 (IQR 24.98–30.86) and 30.6% (*n* = 150) of all patients had a BMI greater than 30.

**Fig. 2:**
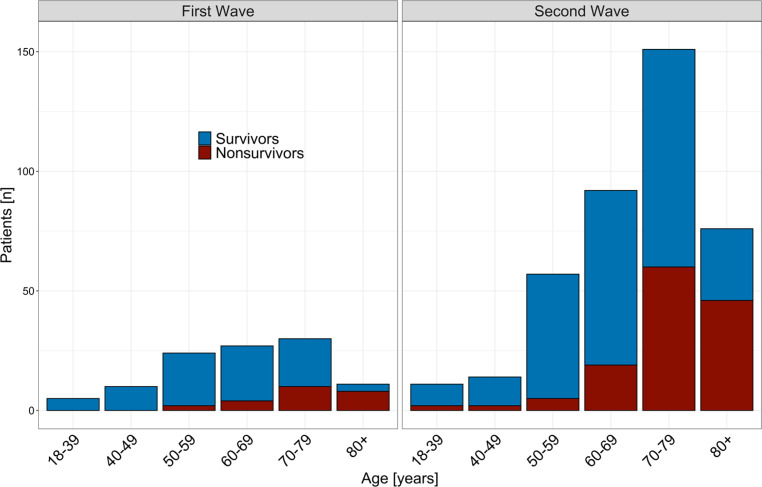
Age and hospital mortality: age distribution of hospital survivors and nonsurvivors in the first and second wave

Patients were significantly older in the second period (64 years, IQR 54–74 years vs. 72 years, IQR 62–78 years, *p* < 0.001, Fig. 2).

The most common comorbidities were hypertension, cardiovascular diseases, diabetes mellitus and renal comorbidities and were relatively similar in both periods. The same applies to other baseline characteristics and risk factors (Table 1).

### Treatment

A total of 274 (54.0%) patients required invasive mechanical ventilation (IMV) for a median duration of 13 days (IQR 7–22 days). Differences in patients treated with or without IMV are shown in the ESM Tables S4–6. In patients requiring IMV, AKI was significantly more frequent and ICU as well as hospital lengths of stay (LOS) were significantly longer (ESM Table S6). Additionally, ICU (35.8% [*n* = 98/274] vs. 17.2% [*n* = 40/233], *p* < 0.001) and hospital mortality (39.1% [*n* = 107/274] vs. 21.5% [*n* = 50/233], *p* < 0.001) were higher compared to patients without IMV. As supportive measure for ARDS (Acute Respiratory Distress Syndrome) prone positioning and neuromuscular blockade were used in 46.2% (*n* = 234) and 22.4% (*n* = 113) of patients, respectively. Of the patients 54.3% (*n* = 274) needed vasopressor therapy during their stay in the ICU and 20 patients (3.9%) required ECMO treatment. Patients were more frequently treated with corticosteroids in the second period (29.1% [*n* = 30/103] vs. 88.3% [*n* = 354/401], *p* < 0.001]).

**Fig. 3:**
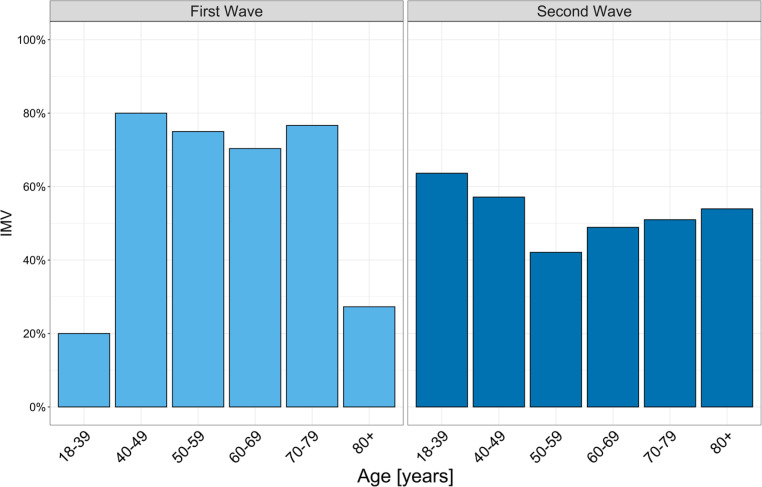
Age and invasive mechanical ventilation (IMV): frequency of IMV in different age groups in the first and second wave

The rate of patients on IMV was significantly higher during the first period (67.3% [*n* = 72] vs. 50.5% [*n* = 202], *p* = 0.003, Fig. 3) and the median duration was significantly longer (15 days, IQR [11–24 days vs 11 days, IQR 6–22 days, *p* = 0.014). By contrast the number of patients with NIV or HFNC was higher during the second period (Table 2).

**Table 2:** Treatment of 508 critically ill COVID-19 patients stratified by two waves [4]

	Overall	First wave	Second wave	*P* value
*n*	508	107	401	–
*IMV (%)*	274 (54.0)	72 (67.3)	202 (50.5)	0.003
*NIV before IMV (%)*	202 (74.8)	52 (77.6)	150 (73.9)	0.656
*HFNC before IMV (%)*	114 (42.2)	15 (22.4)	99 (48.8)	< 0.001
*NIV (never IMV) (%)*	194 (82.9)	28 (80.0)	166 (83.4)	0.801
*HFNC (never IMV) (%)*	123 (52.8)	9 (26.5)	114 (57.3)	0.002
*Prone positioning (%)*	234 (46.2)	58 (54.2)	176 (44.0)	0.076
*Neuromuscular blockade (%)*	–	–	–	< 0.001
– No neuromuscular blockade	393 (77.7)	71 (66.4)	322 (80.7)	–
– Intermittent neuromuscular blockade	99 (19.6)	26 (24.3)	73 (18.3)	–
– Continuous neuromuscular blockade	14 (2.8)	10 (9.3)	4 (1.0)	–
*Vasopressors (%)*	274 (54.3)	74 (69.2)	200 (50.3)	0.001
*RRT (%)*	69 (13.6)	21 (19.6)	48 (12.0)	0.061
*Vv-ECMO (%)*	20 (3.9)	6 (5.6)	14 (3.5)	0.475
*Days on IMV, median (IQR)*	13.00 (7.00–22.00)	15.00 (10.75–24.00)	11.00 (6.00–22.00)	0.014
*Days on NIV, median (IQR)*	3.00 (1.00–6.00)	3.00 (1.00–6.00)	3.00 (1.00–7.00)	0.563
*Days on HFNC, median (IQR)*	3.00 (1.00–6.00)	1.00 (1.00–1.25)	3.00 (1.00–6.00)	< 0.001
*Days with prone positioning, median (IQR)*	3.00 (2.00–6.00)	4.00 (2.00–5.75)	3.00 (2.00–6.00)	0.611
*Days on RRT, median (IQR)*	8.00 (3.00–23.00)	11.00 (3.00–26.00)	6.00 (2.00–18.75)	0.254
*Days on ECMO, median (IQR)*	23.50 (13.50–29.25)	12.00 (11.25–14.25)	26.50 (21.00–29.75)	0.032
*Corticosteroids (%)*	384 (76.2)	30 (29.1)	354 (88.3)	< 0.001

The 107 patients from the first wave have been previously reported [4].

*IQR* interquartile range, *IMV* invasive mechanical ventilation, *NIV* noninvasive ventilation, *HFNC* high flow nasal cannula, *RRT* renal replacement therapy, *vv-ECMO* veno-venous extracorporeal membrane oxygenation, *NA* not available

Like the rates of IMV, the incidence of AKI of all KDIGO stages and correspondingly the requirement of RRT (19.6% [*n* = 21] vs. 12.0% [*n* = 48], *p* = 0.061) has changed over time and was lower in the second period.

### Patient outcome

Overall ICU and hospital mortality was 27.4% (*n* = 139) and 31.1%, respectively (*n* = 158, Table 3). Critically ill patients, who died in hospital were significantly older (77 years, IQR 71–81 years vs. 66 years, IQR 57–75 years, *p* < 0.001, Fig. 2). The overall number of comorbidities was also significantly higher in patients who died in hospital (ESM Table S7). The times from symptom onset to hospital (5 days, IQR 3–8 days vs. 7 days, IQR 4–10 days, *p* < 0.001) and ICU admission (7 days,IQR 4–10 days vs. 9 days, IQR 6–12 days, *p* = 0.001) were significantly shorter and rates of IMV (68.2% [*n* = 107] vs. 47.7% [*n* = 167], *p* < 0.001), prone positioning (58.9% [*n* = 93] vs. 40.4% [*n* = 141], *p* < 0.001) and vasopressor use (75.5% [*n* = 117] vs. 44.9% [*n* = 157], *p* < 0.001) were significantly higher in hospital nonsurvivors (ESM Tables S7–S9).

**Table 3:** Outcome of 508 critically ill COVID-19 patients stratified by two waves [4]

	Overall	First wave	Second wave	*P* value
*n*	508	107	401	–
*Death in ICU (%)*	139 (27.4)	23 (21.5)	116 (28.9)	0.159
*Death in hospital (%)*	158 (31.1)	24 (22.4)	134 (33.4)	0.039
*ICU LOS, days median (IQR)*	11.00 (5.00–22.00)	18.00 (5.00–31.50)	10.00 (5.00–18.00)	< 0.001
*Hospital LOS, days median (IQR)*	21.00 (13.00–35.00)	27.00 (14.50–41.50)	20.00 (13.00–33.75)	0.012
*AKI (%)*				< 0.001
No AKI	343 (68.1)	55 (51.4)	288 (72.5)	–
KDIGO I	52 (10.3)	16 (15.0)	36 (9.1)	–
KDIGO II	31 (6.2)	9 (8.4)	22 (5.5)	–
KDIGO III	78 (15.5)	27 (25.2)	51 (12.8)	–
**Treatment limitations (%)**	137 (27.0)	26 (24.3)	111 (27.7)	0.563
– No CPR (%)	107 (21.1)	22 (20.6)	85 (21.2)	0.992
– No IMV (%)	56 (11.0)	10 (9.3)	46 (11.5)	0.653
– No ECMO (%)	87 (17.1)	17 (15.9)	70 (17.5)	0.812
– Other (%)	71 (14.0)	13 (12.1)	58 (14.5)	0.648
– Best supportive care (%)	75 (14.8)	13 (12.1)	62 (15.5)	0.481

The 107 patients from the first wave have been previously reported [4].

*IQR* interquartile range, *ICU* intensive care unit, *AKI* acute kidney injury, *KDIGO* kidney disease: improving global outcomes, *LOS* length of stay, *CPR* cardiopulmonary resuscitation, *IMV* invasive mechanical ventilation, *ECMO* extracorporeal membrane oxygenation

**Fig. 4:**
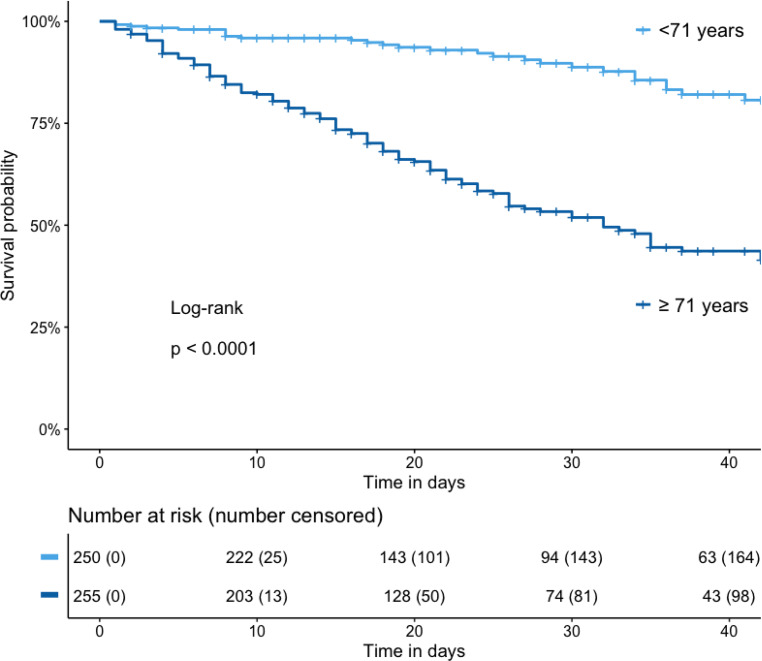
Kaplan-Meier survival curve and number at risk table: overall cohort grouped according to median age in < 71 years and ≥ 71 years; number censored: cumulative number of patients discharged alive from the hospital or lost to follow-up

As illustrated by the Kaplan-Meier analysis, a higher age (> 71 years) was significantly associated with impaired outcome (Fig. 4).

To further assess clinically relevant predictors of hospital mortality, we included age, sex, period of presentation, the number of comorbidities, smoking habits, frailty, if patients came from a nursing home and the time from symptom onset to ICU admission in a logistic regression analysis. Age, the number of comorbidities and the frailty remained significant predictors of hospital mortality after adjustment for other baseline characteristics in the multivariate model (Table 4).

**Table 4:** Logistic regression analysis for prediction of hospital mortality

	Univariate analysis		Multivariate analysis	
	OR (95% CI)	*P* value	OR (95% CI)	*P* value
Age	1.09 (1.07–1.12)	**<** **0.001**	1.09 (1.06–1.12)	**<** **0.001**
Sex (male)	0.85 (0.57–1.29)	0.45	–	–
Period of presentation (second wave)	1.74 (1.07–2.91)	**0.03**	1.27 (0.70–2.24)	0.47
Number of comorbidities	1.41 (1.24–1.60)	**<** **0.001**	1.20 (1.03–1.38)	**0.02**
Active smoking	1.21 (0.61–2.30)	0.58	–	–
Previous smoking	1.10 (0.70–1.71)	0.66	–	–
Frailty	3.44 (2.10–5.68)	**<** **0.001**	1.92 (1.09–3.41)	**0.02**
Time from symptom onset to ICU admission	0.95 (0.91–0.99)	**0.03**	0.98 (0.94–1.03)	0.45
Nursing home	2.26 (0.76–6.71)	0.13	–	–

*OR* odds ratio, *CI* confidence interval, *ICU* intensive care unit

The ICU mortality did not change significantly between the two periods; however, in the first period, hospital mortality was significantly lower (Table 3). Patients stayed longer in the hospital (27 day, IQR 15–42 days vs. 20 days, IQR 13–34 days, *p* = 0.012) and in the ICU (18 days, IQR 5–32 days vs. 10 days, IQR 5–18 days, *p* < 0.001) than in the second period. The rate of patients with a documented treatment limitation was relatively similar during both periods (24.3% [*n* = 26] vs. 27.7% [*n* = 111], *p* = 0.563). After 1:1 propensity score matching, a better balance of baseline characteristics was established (Supplemental Table 11). While in univariate analysis there was a significant association between the period of presentation and hospital mortality, no significance was found in the analysis of the matched cohort (Supplemental Table 12).

## Discussion

This was a prospective observational register study of 508 critically ill patients treated at an ICU in Tyrol (Austria), between 9 March 2020 and 22 February 2021 with confirmed SARS-CoV‑2 infections. About four times more patients had to be treated in the ICU during the second period compared to the first one. While most baseline characteristics were similar, patients were significantly older in the second wave. Patients of the second period required less IMV, RRT and vasopressors, resulting in drastically reduced LOS. Despite that, hospital mortality increased, albeit being still lower than or similar to rates reported from other regions of Europe [10–12]. Age, number of comorbidities and frailty have shown to be independent predictors of hospital mortality.

The most remarkable difference between the two periods is the higher number of patients in the second wave (401 vs. 107), with a new maximum of the peak in ICU occupancy with 82 patients (Fig. 1). These numbers correspond to the dynamics of the pandemic in Austria [1]. As mentioned in our previous paper we had established an ICU resource management, which avoided overcrowding of ICU and healthcare system decompensation. This was also successful during the second wave; however, to maintain unrestricted access to the ICU, improvements in the treatment of critically ill COVID-19 patients were necessary.

Major progress has been made in ventilation treatment, as reflected in lower rates and duration of IMV in the second period. Corresponding to the reduction of IMV, the number of patients who only required NIV or HFNC was higher during the second period (Table 2). A similar decrease of IMV use has been reported from other cohorts [13, 14]. Several factors may be responsible for this finding. First of all the change of the rate of IMV corresponds to the change of recommendations regarding timing of intubation [15]. High rates of mechanical ventilation in critically ill patients and a rapid deterioration have been reported from China [16] and Italy [10]. Therefore, in the beginning, early intubation was considered beneficial not only for ICU staff due to less aerosol exposure but also for the patients. Respiratory treatment with NIV and HFNC have been proven to avoid invasive ventilation in comparison to a standard oxygen mask [17]. Although NIV and HFNC are important and might have the ability to protect patients from IMV, late failure of NIV may increase mortality in these patients [18]; however, randomized controlled trials are necessary to answer the question of optimal timing of intubation. Additionally, the significantly increased use of corticosteroids in the second wave, after the results of the RECOVERY trial were published, may have contributed to a reduced need and duration of invasive ventilation [6, 19].

Interestingly, the rate of AKI was lower in all KDIGO stages in the second wave (Table 3). Lung-kidney interactions seem to play an important role in critically ill patients [20] and IMV is an important risk factor for AKI. Therefore, the lower rate of IMV may be also responsible for the decline in the AKI incidence [21]. These changes are also reflected in the lower rate of RRT in the second wave, which may be an important factor influencing ICU LOS. Since the immune response plays an important role in the pathophysiology of AKI [22] and the RECOVERY trial showed a reduced rate of RRT in the dexamethasone group [6], the widespread use of corticosteroids may also have positively influenced these results.

These reduced rates of IMV and RRT were associated with a significantly reduced median ICU LOS by 8 days (18 vs. 10 days, *p* ≤ 0.001); however, this did not end up in a reduced hospital mortality. While ICU mortality did not change significantly, hospital mortality increased. Some studies found improved mortality rates over time, whereas others found no differences [13, 14, 23, 24]; however, comparison to different ICU cohorts may be difficult due to the numerous influencing factors on the ICU population [25]. Additionally, the mortality we found in our first wave was already very low with 22.4% [4]. This remarkably low mortality rate has been discussed in detail [4]. Furthermore, a hospital mortality of 33.4% (second wave) in critically ill patients is still lower or similar compared to other studies [10, 12, 26] and was below the Austrian average. This indicates a successful ICU resource management as was already the case in the first wave.

When correcting for other baseline characteristics in a propensity-matched analysis, there was no significant association between the period of presentation (first or second wave) and hospital mortality. While an influence of the period cannot be completely excluded, other factors seem to be more important. When patient characteristics were analyzed to find possible explanations for the slight increase in hospital mortality, the most important difference between the two groups was an 8‑year increase in median age in the second period (72 years vs. 64 years). In our logistic regression model age was an independent predictor of hospital mortality after adjustment for other baseline characteristics (Table 4). In other studies, age was the strongest predictor for mortality in critically ill patients [27]. This is also supported by the Kaplan-Meier analysis, showing a significant association between older age and impaired hospital survival (Fig. 4).

This raises the question why the median age changed in our cohort. To date, few studies have looked at the differences between the first and second wave of hospitalized and ICU patients in the COVID-19 pandemic [13, 14, 23, 24]; however, no analysis found similar changes of age distribution over time [13, 14, 23, 24]. Our overall median age was relatively high compared to other cohorts from the first wave [10, 16, 28]. A country comparable in terms of overall age distribution such as Germany had similar median age in patients requiring invasive mechanical ventilation [26]. Furthermore, it cannot be ruled out that a change in admission policy over time contributed to the changing age patterns. The increased use of NIV and HFNC may have influenced ICU admissions, especially in old patients. Despite the higher median age, the frequency of treatment limitations remained constant and thus probably did not affect mortality; however, of all patients who died, the majority (70.3%) had a documented treatment limitation.

Factors other than age may also be important. In the logistic regression analysis, the number of comorbidities also remained significant after adjustment for other characteristics and seems to be a relevant risk factor for hospital mortality. The number of comorbidities was higher during the second wave, without reaching significance. While most studies focused on the evaluation of selected comorbidities [10, 12, 26, 28], the overall number of comorbidities might be an even more important indicator of hospital mortality. The third significant risk factor for hospital mortality in our cohort was frailty. Especially in older patients, frailty is an important outcome predictor in critically ill COVID-19 patients [29].

Our study has limitations due to its observational design. Therefore, potential biases from changing strategies over time and other influencing factors exist. Some important information like the severity of ARDS was missing; however SAPS III and SOFA scores at admission were available. We cannot exclude influence from the virus variants of concern; however, the largest period of the second wave was before the appearance of these variants in Austria (1 January 2021).

The strength of our study consists in its multicenter character, which includes both ICUs from peripheral and central university hospitals. Although other cohort studies reached higher patient numbers, the very similar baseline characteristics reflect good comparability. We included all critically ill patients from a region with 750,000 inhabitants.

## Conclusion

Therapeutic strategies for patients with COVID-19 at the ICU improved over time, which lead to a reduction of IMV, AKI and RRT and subsequently to a reduced ICU LOS in the second period. Age is an important predictor of hospital mortality, with a strong influence on outcomes of critically ill COVID-19 patients.

## Supplementary Information

List of COVID-19 ICUs in Tyrol, Supplemental Tables 1–11, Supplemental Figure 1
