# Supplementary material for: Changes in characteristics and outcomes of critically ill COVID-19 patients in Tyrol (Austria) over 1 year
Source: Wien Klin Wochenschr. 2021 Oct 18;133(23-24):1237–47. doi: 10.1007/s00508-021-01945-5 (PMC8521508; doi:10.1007/s00508-021-01945-5)
Supplement: Supplementary file 1 — List of COVID-19 ICUs in Tyrol, Supplemental Tables 1–11, Supplemental Figure 1 [file 508_2021_1945_MOESM1_ESM.pdf]

Electronic Supplementary Material

Changes in characteristics and outcomes of critically ill COVID-19 patients in Tyrol (Austria) over 1 year

List of COVID-19 ICUs in Tyrol

Supplemental Tables 1-11

Supplemental Figure 1

List of COVID-19 intensive care units (ICUs) in Tyrol, Austria who participated in the Tyrolean COVID-19 Intensive Care Registry (Tyrol-CoV-ICU-Reg):

- Medical University Innsbruck, Innsbruck, Austria
  - First wave:
    - Medical ICU, Department of Internal Medicine
    - Neurosurgical ICU, Department of Neurosurgery
    - Transplantation ICU, Department of General and Surgical Intensive Care Medicine/Department of Anesthesia and Critical Care Medicine
    - Recovery room "OZA" (adapted as temporary ICU), Department of General and Surgical Intensive Care Medicine/Department of Anesthesia and Critical Care Medicine
    - Recovery room "KHZ" (adapted as temporary ICU), Department of General and Surgical Intensive Care Medicine/Department of Anesthesia and Critical Care Medicine
    - Pediatric Intensive Care Unit, Department of Pediatrics
  - Second wave:
    - Medical ICU, Department of Internal Medicine
    - Neurosurgical ICU, Department of Neurosurgery
    - Transplantation ICU, Department of General and Surgical Intensive Care Medicine/Department of Anesthesia and Critical Care Medicine
    - General surgery ICU, Department of General and Surgical Intensive Care Medicine/Department of Anesthesia and Critical Care Medicine
    - Cardiac surgery ICU, Department of General and Surgical Intensive Care Medicine/Department of Anesthesia and Critical Care Medicine
    - Pediatric Intensive Care Unit, Department of Pediatrics
- Hospital Hall, Hall, Austria
  - ICU, Department of Anesthesia and Intensive Care Medicine
- Hospital Kufstein, Kufstein, Austria
  - ICU, Department of Anesthesia and Intensive Care Medicine
- Hospital Lienz, Lienz, Austria
  - ICU, Department of Anesthesia and Intensive Care Medicine
- Hospital Reutte, Reutte, Austria
  - ICU, Department of Anesthesia and Intensive Care Medicine

- Hospital Schwaz, Schwaz, Austria
  - ICU, Department of Anesthesia and Critical Care Medicine
- Hospital St. Johann i.T., St. Johann in Tyrol, Austria
  - ICU, Department of Anesthesia and Intensive Care Medicine
- Hospital Zams
  - Medical ICU, Department of Internal Medicine
  - Surgical ICU, Department of Anesthesiology and Critical Care Medicine

Supplemental Table 1 *Baseline characteristics of 4 critically ill COVID-19 pediatric patients*

	Overall
n	4
Sex: male/female (%)	3/1 (75.0/25.0)
Age, years median (IQR)	7.00 (2.25-12.50)
**Comorbidities**	
Hypertension (%)	0 (0)
Cardiovascular (%)	0 (0)
Diabetes (%)	
Prediabetes	0 (0)
DM Type I	0 (0)
DM Type II	0 (0)
DM (other Type)	0 (0)
Renal (%)	0 (0)
Neurological (%)	0 (0)
Liver (%)	0 (0)
Hematological malignancy (%)	0 (0)
Immunosuppression (%)	0 (0)
Nonhematological malignancy (%)	0 (0)
COPD (%)	0 (0)
Asthma (%)	0 (0)
Respiratory - other (%)	0 (0)
Overweight (BMI≥25) (%)	0 (0)
Obesity (BMI≥30) (%)	0 (0)
No known comorbidity (%)	4 (100.0)
Number of comorbidities, n	0.00 (0.00-0.00)
BMI, kg/m² median (IQR)	19.82 (15.12-23.63)
HbA1c, % median (IQR)	5.50 (5.45-5.85)
**Risk factors**	
Active smoking (%)	0 (0)
Previous smoking (%)	0 (0)
Patient from nursing home (%)	0 (0)
Frailty	
Fully independent daily living (%)	2 (66.7)
Pre-frail (%)	0 (0.0)
Frail (%)	1 (33.3)
COVID-19 primary reason for hospital admission (%)	1 (25.0)
COVID-19 typical findings in chest x-ray (%)	2 (50.0)
COVID-19 typical findings in computed tomography (%)	0 (0)
SAPS III, median (IQR)	52.50 (51.25-53.75)
Time from symptom onset to hospital admission, days median (IQR)	5.50 (3.75-6.25)
Time from symptom onset to ICU admission, days median (IQR)	6.00 (4.50-6.50)

IQR – interquartile range, DM – diabetes mellitus, COPD – chronic obstructive pulmonary disease, BMI – body mass index, HbA1c – glycated hemoglobin, SOFA – sequential organ failure assessment, SAPS – simplified acute physiology score, ICU – intensive care unit

Supplemental Table 2 *Treatment of 4 critically ill COVID-19 pediatric patients*

	Overall
n	4
IMV (%)	1 (25.0)
NIV before IMV (%)	1 (100.0)
HFNC before IMV (%)	1 (100.0)
NIV (never IMV) (%)	0 (0)
HFNC (never IMV) (%)	1 (33.3)
Prone Positioning (%)	0 (0)
Neuromuscular blockade (%)	
no neuromuscular blockade	4 (100.0)
Vasopressors (%)	2 (50.0)
RRT (%)	0 (0)
vv-ECMO (%)	0 (0)
Days on IMV, median (IQR)	0.00 (0.00-0.00)
Days on NIV, median (IQR)	4.00 (4.00-4.00)
Days on HFNC, median (IQR)	2.50 (1.75-3.25)
Corticosteroids (%)	3 (75.0)

IQR – interquartile range, IMV – invasive mechanical ventilation, NIV – noninvasive ventilation, HFNC- high flow nasal cannula, RRT – renal replacement therapy, vv-ECMO – veno-venous extracorporeal membrane oxygenation,

Supplemental Table 3 *Outcome of 4 critically ill COVID-19 pediatric patients*

	Overall
n	4
Death in ICU (%)	0 (0)
Death in Hospital (%)	0 (0)
ICU LOS, days median (IQR)	4.00 (3.75-4.00)
Hospital LOS, days median (IQR)	7.00 (5.50-8.50)
AKI (%)	
No AKI	4 (100.0)
KDIGO I	0 (0)
KDIGO II	0 (0)
KDIGO III	0 (0)
Treatment limitations (%)	0 (0)
No CPR (%)	0 (0)
No IMV (%)	0 (0)
No ECMO (%)	0 (0)
other (%)	0 (0)
Best supportive care (%)	0 (0)

IQR – interquartile range, ICU – intensive care unit, AKI – acute kidney injury, KDIGO – kidney disease: improving global outcomes, LOS – length of stay, CPR – cardiopulmonary resuscitation

Supplemental Table 4 *Baseline characteristics of 508 critically ill COVID-19 patients grouped by receipt of invasive mechanical ventilation*

	Overall	No IMV	IMV	*P* value
n	508	233	274	
Sex: male/female (%)	356/152 (70.1/29.9)	159/74 (68.2/31.8)	197/77 (71.9/28.1)	0.424
Age, years median (IQR)	71.00 (60.00-78.00)	70.00 (61.00-78.00)	71.00 (60.00-78.00)	0.855
**Comorbidities**				
Hypertension (%)	327 (64.4)	155 (66.5)	171 (62.4)	0.384
Cardiovascular (%)	217 (42.7)	101 (43.3)	116 (42.3)	0.889
Diabetes (%)				0.680
Prediabetes	15 (3.0)	8 (3.4)	7 (2.6)	
DM Type I	5 (1.0)	3 (1.3)	2 (0.7)	
DM Type II	114 (22.4)	56 (24.0)	58 (21.2)	
DM (other Type)	1 (0.2)	0 (0.0)	1 (0.4)	
Renal (%)	108 (21.3)	46 (19.7)	62 (22.6)	0.495
Neurological (%)	75 (14.8)	34 (14.6)	41 (15.0)	1.000
Liver (%)	38 (7.5)	16 (6.9)	22 (8.0)	0.744
Hematological malignancy (%)	26 (5.1)	16 (6.9)	10 (3.6)	0.151
Immunosuppression (%)	36 (7.1)	15 (6.4)	21 (7.7)	0.717
Nonhematological malignancy (%)	40 (7.9)	20 (8.6)	20 (7.3)	0.701
COPD (%)	70 (13.8)	38 (16.3)	32 (11.7)	0.169
Asthma (%)	21 (4.1)	9 (3.9)	12 (4.4)	0.946
Respiratory - other (%)	41 (8.1)	22 (9.4)	19 (6.9)	0.385
Overweight (BMI≥25) (%)	363 (74.1)	162 (73.3)	200 (74.6)	0.819
Obesity (BMI≥30) (%)	150 (30.6)	72 (32.6)	77 (28.7)	0.411
No known comorbidity (%)	47 (9.6)	18 (8.2)	29 (10.8)	0.407
Number of comorbidities, n median (IQR)	2.00 (1.00-4.00)	3.00 (1.00-4.00)	2.00 (1.00-4.00)	0.189
BMI, kg/m² median (IQR)	27.52 (24.98-30.86)	27.49 (24.96-31.05)	27.51 (24.98-30.75)	0.863
HbA1c, % median (IQR)	6.30 (5.90-6.80)	6.29 (5.90-7.00)	6.30 (5.90-6.80)	0.803
**Risk factors**				
Active smoking (%)	43 (9.9)	18 (8.7)	24 (10.5)	0.629
Previous smoking (%)	116 (28.2)	52 (27.5)	63 (28.4)	0.933
Patient from nursing home (%)	14 (2.8)	7 (3.0)	7 (2.6)	0.963
Frailty				0.009
Fully independent daily living (%)	422 (84.4)	182 (79.1)	239 (88.8)	
Pre-frail (%)	70 (14.0)	42 (18.3)	28 (10.4)	
Frail (%)	8 (1.6)	6 (2.6)	2 (0.7)	
COVID-19 primary reason for hospital admission (%)	422 (83.1)	185 (79.4)	236 (86.1)	0.058
COVID-19 typical findings in chest x-ray (%)	458 (92.0)	198 (88.0)	259 (95.2)	0.005
COVID-19 typical findings in computed tomography (%)	256 (87.7)	102 (87.2)	153 (87.9)	0.993
SOFA Score, median (IQR)	5.00 (4.00-8.00)	4.00 (3.00-6.00)	7.00 (4.00-9.00)	<0.001
SAPS III Score, median (IQR)	56.00 (49.00-64.00)	52.00 (47.00-62.00)	58.00 (51.00-66.00)	<0.001
Time from symptom onset to hospital admission, days median (IQR)	6.00 (3.00-9.00)	6.00 (3.00-9.00)	7.00 (4.00-10.00)	0.048
Time from symptom onset to ICU admission, days median (IQR)	8.00 (5.00-11.00)	8.00 (5.00-11.00)	8.00 (6.00-12.00)	0.067

IQR – interquartile range, DM – diabetes mellitus, COPD – chronic obstructive pulmonary disease, BMI – body mass index, HbA1c – glycated hemoglobin, SOFA – sequential organ failure assessment, SAPS – simplified acute physiology score, ICU – intensive care unit

Supplemental Table 5 *Treatment grouped by receipt of invasive mechanical ventilation*

	Overall	No IMV	IMV	*P* value
n	508	233	274	
IMV (%)	274 (54.0)	0 (0.0)	274 (100.0)	<0.001
NIV before IMV (%)	202 (74.8)	0 (NA)	202 (75.1)	NA
HFNC before IMV (%)	114 (42.2)	0 (NA)	114 (42.4)	NA
NIV (never IMV) (%)	194 (82.9)	194 (83.3)	0 (NA)	NA
HFNC (never IMV) (%)	123 (52.8)	123 (53.0)	0 (NA)	NA
Prone Positioning (%)	234 (46.2)	30 (12.9)	203 (74.1)	<0.001
Neuromuscular blockade (%)				<0.001
no neuromuscular blockade	393 (77.7)	231 (100.0)	162 (59.1)	
intermittent neuromuscular blockade	99 (19.6)	0 (0.0)	98 (35.8)	
continuous neuromuscular blockade	14 (2.8)	0 (0.0)	14 (5.1)	
Vasopressors (%)	274 (54.3)	33 (14.2)	241 (88.6)	<0.001
RRT (%)	69 (13.6)	5 (2.2)	64 (23.4)	<0.001
vv-ECMO (%)	20 (3.9)	0 (0.0)	20 (7.3)	<0.001
Days on IMV, median (IQR)	13.00 (7.00-22.00)	NA	13.00 (7.00-22.00)	NA
Days on NIV, median (IQR)	3.00 (1.00-6.00)	5.00 (2.00-7.00)	2.00 (1.00-5.00)	<0.001
Days on NHF, median (IQR)	3.00 (1.00-6.00)	5.00 (2.00-7.00)	2.00 (1.00-3.00)	<0.001
Days with Prone Positioning, median (IQR)	3.00 (2.00-6.00)	3.00 (1.00-4.00)	3.00 (2.00-6.50)	0.061
Days on RRT, median (IQR)	8.00 (3.00-23.00)	1.00 (1.00-1.00)	10.00 (3.00-24.25)	0.001
Days on ECMO, median (IQR)	23.50 (13.50-29.25)	NA	23.50 (13.50-29.25)	NA
Corticosteroids (%)	384 (76.2)	175 (76.1)	208 (76.2)	1.000

IQR – interquartile range, IMV – invasive mechanical ventilation, NIV – noninvasive ventilation, HFNC – high flow nasal cannula, RRT – renal replacement therapy, vv-ECMO – veno-venous extracorporeal membrane oxygenation, NA – Not available

Supplemental Table 6 *Outcome grouped by receipt of invasive mechanical ventilation*

	Overall	No IMV	IMV	*P* value
n	508	233	274	
Death in ICU (%)	139 (27.4)	40 (17.2)	98 (35.8)	<0.001
Death in Hospital (%)	158 (31.1)	50 (21.5)	107 (39.1)	<0.001
Hospital LOS, days median (IQR)	21.00 (13.00-35.00)	15.00 (9.00-23.00)	28.00 (19.00-44.00)	<0.001
ICU LOS, days median (IQR)	11.00 (5.00-22.00)	6.00 (3.00-9.00)	20.00 (13.00-32.00)	<0.001
AKI (%)				<0.001
No AKI	343 (68.1)	208 (89.7)	134 (49.4)	
KDIGO I	52 (10.3)	11 (4.7)	41 (15.1)	
KDIGO II	31 (6.2)	5 (2.2)	26 (9.6)	
KDIGO III	78 (15.5)	8 (3.4)	70 (25.8)	
**Treatment limitations (%)**	137 (27.0)	58 (24.9)	79 (28.8)	0.371
No CPR (%)	107 (21.1)	48 (20.6)	59 (21.5)	0.883
No IMV (%)	56 (11.0)	51 (21.9)	5 (1.8)	<0.001
No ECMO (%)	87 (17.1)	37 (15.9)	50 (18.2)	0.557
other (%)	71 (14.0)	33 (14.2)	38 (13.9)	1.000
Best supportive care (%)	75 (14.8)	27 (11.6)	48 (17.5)	0.080

IQR – interquartile range, ICU- intensive care unit, AKI – acute kidney injury, KDIGO – kidney disease: improving global outcomes, LOS – length of stay, CPR – cardiopulmonary resuscitation, IMV – invasive mechanical ventilation, ECMO - extracorporeal membrane oxygenation

Supplemental Table 7 *Baseline characteristics grouped by hospital death*

	Overall	Survivors	Nonsurvivors	*P* value
n	508	350	158	
Sex: male/female (%)	356/152 (70.1/29.9)	242/108 (69.1/30.9)	114/44 (72.2/27.8)	0.561
Age, years median (IQR)	71.00 (60.00-78.00)	66.00 (57.00-75.00)	77.00 (71.00-81.00)	<0.001
**Comorbidities**				
Hypertension (%)	327 (64.4)	212 (60.6)	115 (72.8)	0.010
Cardiovascular (%)	217 (42.7)	125 (35.7)	92 (58.2)	<0.001
Diabetes (%)				0.154
Prediabetes	15 (3.0)	6 (1.7)	9 (5.7)	
DM Type I	5 (1.0)	3 (0.9)	2 (1.3)	
DM Type II	114 (22.4)	80 (22.9)	34 (21.5)	
DM (other Type)	1 (0.2)	1 (0.3)	0 (0.0)	
Renal (%)	108 (21.3)	55 (15.7)	53 (33.5)	<0.001
Neurological (%)	75 (14.8)	43 (12.3)	32 (20.3)	0.027
Liver (%)	38 (7.5)	20 (5.7)	18 (11.4)	0.038
Hematological malignancy (%)	26 (5.1)	12 (3.4)	14 (8.9)	0.019
Immunosuppression (%)	36 (7.1)	24 (6.9)	12 (7.6)	0.910
Nonhematological malignancy (%)	40 (7.9)	23 (6.6)	17 (10.8)	0.143
COPD (%)	70 (13.8)	40 (11.4)	30 (19.0)	0.032
Asthma (%)	21 (4.1)	17 (4.9)	4 (2.5)	0.328
Respiratory - other (%)	41 (8.1)	26 (7.4)	15 (9.5)	0.539
Overweight (BMI≥25) (%)	363 (74.1)	254 (75.8)	109 (70.3)	0.238
Obesity (BMI≥30) (%)	150 (30.6)	109 (32.5)	41 (26.5)	0.210
No known comorbidity (%)	47 (9.6)	43 (12.8)	4 (2.6)	0.001
Number of comorbidities, n median (IQR)	2.00 (1.00-4.00)	2.00 (1.00-3.00)	3.00 (2.00-4.00)	<0.001
BMI, kg/m² median (IQR)	27.52 (24.98-30.86)	27.64 (25.10-31.12)	27.34 (24.52-30.56)	0.351
HbA1c, % median (IQR)	6.30 (5.90-6.80)	6.30 (5.80-6.80)	6.30 (5.90-6.93)	0.535
**Risk factors**				
Active smoking (%)	43 (9.9)	28 (9.4)	15 (10.8)	0.785
Previous smoking (%)	116 (28.2)	78 (28.1)	38 (28.4)	1.000
Patient from nursing home (%)	14 (2.8)	7 (2.0)	7 (4.5)	0.213
Frailty				<0.001
Fully independent daily living (%)	422 (84.4)	311 (89.9)	111 (72.1)	
Pre-frail (%)	70 (14.0)	33 (9.5)	37 (24.0)	
Frail (%)	8 (1.6)	2 (0.6)	6 (3.9)	
COVID-19 primary reason for hospital admission (%)	422 (83.1)	289 (82.6)	133 (84.2)	0.750
COVID-19 typical findings in chest x-ray (%)	458 (92.0)	311 (90.7)	147 (94.8)	0.160
COVID-19 typical findings in computed tomography (%)	256 (87.7)	180 (88.2)	76 (86.4)	0.801
SOFA Score, median (IQR)	5.00 (4.00-8.00)	4.00 (3.00-7.00)	7.00 (4.00-9.00)	<0.001
SAPS III Score, median (IQR)	56.00 (49.00-64.00)	53.00 (47.00-60.00)	62.00 (55.00-70.00)	<0.001
Time from symptom onset to hospital admission, days median (IQR)	6.00 (3.00-9.00)	7.00 (4.00-10.00)	5.00 (3.00-8.00)	<0.001
Time from symptom onset to ICU admission, days median (IQR)	8.00 (5.00-11.00)	9.00 (6.00-12.00)	7.00 (4.00-10.00)	0.001

IQR – interquartile range, DM – diabetes mellitus, COPD – chronic obstructive pulmonary disease, BMI – body mass index, HbA1c – glycated hemoglobin, SOFA – sequential organ failure assessment, SAPS – simplified acute physiology score, ICU – intensive care unit

Supplemental Table 8 *Treatment grouped by hospital death*

	Overall	Survivors	Nonsurvivors	*P* value
n	508	350	158	
IMV (%)	274 (54.0)	167 (47.7)	107 (68.2)	<0.001
NIV before IMV (%)	202 (74.8)	120 (73.6)	82 (76.6)	0.678
HFNC before IMV (%)	114 (42.2)	70 (42.9)	44 (41.1)	0.864
NIV (never IMV) (%)	194 (82.9)	150 (82.0)	44 (86.3)	0.608
HFNC (never IMV) (%)	123 (52.8)	96 (52.7)	27 (52.9)	1.000
Prone Positioning (%)	234 (46.2)	141 (40.4)	93 (58.9)	<0.001
Neuromuscular blockade (%)				<0.001
no neuromuscular blockade	393 (77.7)	288 (82.8)	105 (66.5)	
intermittent neuromuscular blockade	99 (19.6)	53 (15.2)	46 (29.1)	
continuous neuromuscular blockade	14 (2.8)	7 (2.0)	7 (4.4)	
Vasopressors (%)	274 (54.3)	157 (44.9)	117 (75.5)	<0.001
RRT (%)	69 (13.6)	32 (9.2)	37 (23.6)	<0.001
vv-ECMO (%)	20 (3.9)	12 (3.4)	8 (5.1)	0.532
Days on IMV, median (IQR)	13.00 (7.00-22.00)	12.00 (7.00-21.00)	14.00 (6.25-23.00)	0.844
Days on NIV, median (IQR)	3.00 (1.00-6.00)	4.00 (2.00-7.00)	2.00 (1.00-4.00)	<0.001
Days on NHF, median (IQR)	3.00 (1.00-6.00)	3.00 (1.00-6.00)	2.00 (1.00-3.50)	0.006
Days with Prone Positioning, median (IQR)	3.00 (2.00-6.00)	3.00 (1.00-5.00)	4.00 (2.00-8.00)	0.003
Days on RRT, median (IQR)	8.00 (3.00-23.00)	14.00 (5.25-25.25)	4.00 (2.00-11.00)	0.033
Days on ECMO, median (IQR)	23.50 (13.50-29.25)	18.00 (11.75-29.75)	26.50 (23.25-28.50)	0.279
Corticosteroids (%)	384 (76.2)	255 (73.5)	129 (82.2)	0.045

IQR – interquartile range, IMV – invasive mechanical ventilation, NIV – noninvasive ventilation, HFNC- high flow nasal cannula, RRT – renal replacement therapy, vv-ECMO – veno-venous extracorporeal membrane oxygenation

Supplemental Table 9 *Outcome grouped by hospital death*

	Overall	Survivors	Nonsurvivors	*P* value
n	508	350	158	
Death in ICU (%)	139 (27.4)	0 (0.0)	139 (88.0)	<0.001
Death in Hospital (%)	158 (31.1)	0 (0.0)	158 (100.0)	<0.001
Hospital LOS, days median (IQR)	21.00 (13.00-35.00)	23.00 (15.00-40.00)	17.00 (8.00-27.00)	<0.001
ICU LOS, days median (IQR)	11.00 (5.00-22.00)	11.00 (5.00-22.00)	10.00 (4.00-22.00)	0.478
AKI (%)				<0.001
No AKI	343 (68.1)	269 (77.3)	74 (47.4)	
KDIGO I	52 (10.3)	36 (10.3)	16 (10.3)	
KDIGO II	31 (6.2)	16 (4.6)	15 (9.6)	
KDIGO III	78 (15.5)	27 (7.8)	51 (32.7)	
**Treatment limitations (%)**	137 (27.0)	26 (7.4)	111 (70.3)	<0.001
No CPR (%)	107 (21.1)	22 (6.3)	85 (53.8)	<0.001
No IMV (%)	56 (11.0)	13 (3.7)	43 (27.2)	<0.001
No ECMO (%)	87 (17.1)	20 (5.7)	67 (42.4)	<0.001
other (%)	71 (14.0)	12 (3.4)	59 (37.3)	<0.001
Best supportive care (%)	75 (14.8)	1 (0.3)	74 (46.8)	<0.001

IQR – interquartile range, ICU – intensive care unit, AKI – acute kidney injury, KDIGO – kidney disease: improving global outcomes, LOS – length of stay, CPR – cardiopulmonary resuscitation, IMV – invasive mechanical ventilation, ECMO - extracorporeal membrane oxygenation

Supplemental Table 10 *Baseline characteristics before and after propensity score matching*

	Before propensity score matching			After propensity score matching		
	First wave (n=105)	Second wave (n=378)	Standardized Difference	First wave (n=105)	Second wave (n=105)	Standardized Difference
Sex: male/female (%)	75/30 (71.4/28.6)	262/116 (69.3/30.7)	0.046	75/30 (71.4/28.6)	74/31 (70.5/29.5)	0.021
Age, years median (IQR)	64.00 (54.00-75.00)	72.00 (61.00-78.00)	0.394	64.00 (54.00-75.00)	68.00 (56.00-76.00)	0.050
**Comorbidities**						
Hypertension (%)	71 (67.6)	243 (64.3)	0.070	71 (67.6)	75 (71.4)	0.083
Cardiovascular (%)	45 (42.9)	159 (42.1)	0.016	45 (42.9)	55 (52.4)	0.192
Diabetes (%)			0.284			0.181
Prediabetes	2 (1.9)	12 (3.2)		85 (81.0)	90 (85.7)	
DM Type I	1 (1.0)	4 (1.1)		2 (1.9)	2 (1.9)	
DM Type II	16 (15.2)	92 (24.3)		1 (1.0)	1 (1.0)	
DM (other Type)	1 (1.0)	0 (0.0)		16 (15.2)	12 (11.4)	
Renal (%)	21 (20.0)	81 (21.4)	0.035	21 (20.0)	23 (21.9)	0.047
Neurological (%)	11 (10.5)	60 (15.9)	0.0160	11 (10.5)	11 (10.5)	<0.001
Liver (%)	7 (6.7)	28 (7.4)	0.029	7 (6.7)	4 (3.8)	0.129
Hematological malignancy (%)	3 (2.9)	21 (5.6)	0.135	3 (2.9)	7 (6.7)	0.180
Immunosuppression (%)	11 (10.5)	25 (6.6)	0.138	11 (10.5)	10 (9.5)	0.032
Nonhematological malignancy (%)	5 (4.8)	29 (7.7)	0.121	5 (4.8)	5 (4.8)	<0.001
COPD (%)	14 (13.3)	51 (13.5)	0.5	14 (13.3)	13 (12.4)	0.028
Asthma (%)	7 (6.7)	13 (3.4)	0.148	7 (6.7)	5 (4.8)	0.082

Respiratory - other (%)	13 (12.4)	27 (7.1)	0.177	13 (12.4)	12 (11.4)	0.029
No known comorbidity (%)	15 (14.3)	34 (9.0)	0.166	15 (14.3)	9 (8.6)	0.180
BMI, median (IQR))	27.18 (25.13-29.76)	27.77 (25.10-30.86)	0.174	27.18 (25.13-29.76)	27.47 (24.98-29.63)	0.014
**Risk factors**						
Active smoking (%)	11 (10.5)	30 (7.9)	0.088	11 (10.5)	12 (11.4)	0.030
Previous smoking (%)	25 (23.8)	86 (22.8)	0.025	25 (23.8)	26 (24.8)	0.022
Patient from nursing home (%)	0 (0.0)	13 (3.4)	0.267	105 (100.0)	105 (100.0)	<0.001
Frailty	15 (14.3)	57 (15.1)	0.022	15 (14.3)	13 (12.4)	0.056
COVID-19 primary reason for hospital admission (%)	90 (85.7)	318 (84.1)	0.044	90 (85.7)	84 (80.0)	0.152
SAPS III Score, median (IQR)	56.00 (49.00-64.00)	55.00 (49.00-64.00)	0.033	56.00 (49.00-64.00)	56.00 (48.00-68.00)	0.055
Time from symptom onset to ICU admission, days median (IQR))	8.00 (6.00-11.00)	8.82 (6.00-11.00)	0.095	8.00 (6.00-11.00)	8.82 (7.00-10.00)	0.057

IQR – interquartile range, DM – diabetes mellitus, COPD – chronic obstructive pulmonary disease, BMI – body mass index, HbA1c – glycated hemoglobin, SOFA – sequential organ failure assessment, SAPS – simplified acute physiology score, ICU – intensive care unit

***Supplemental Table 11** (Conditional) logistic regression analysis of the propensity matched cohort for hospital mortality*

	Logistic Regression of unmatched cohort		*Conditional logistic regression of propensity matched cohort*	
	OR (95% CI)	*P* value	OR (95% CI)	*P* value
Period of Presentation (Ref: Second Wave)	0.59 (0.35-0.96)	0.037	0.66 (0.37-1.17)	0 .152

OR – odds ratio, CI – confidence interval, Ref – reference category

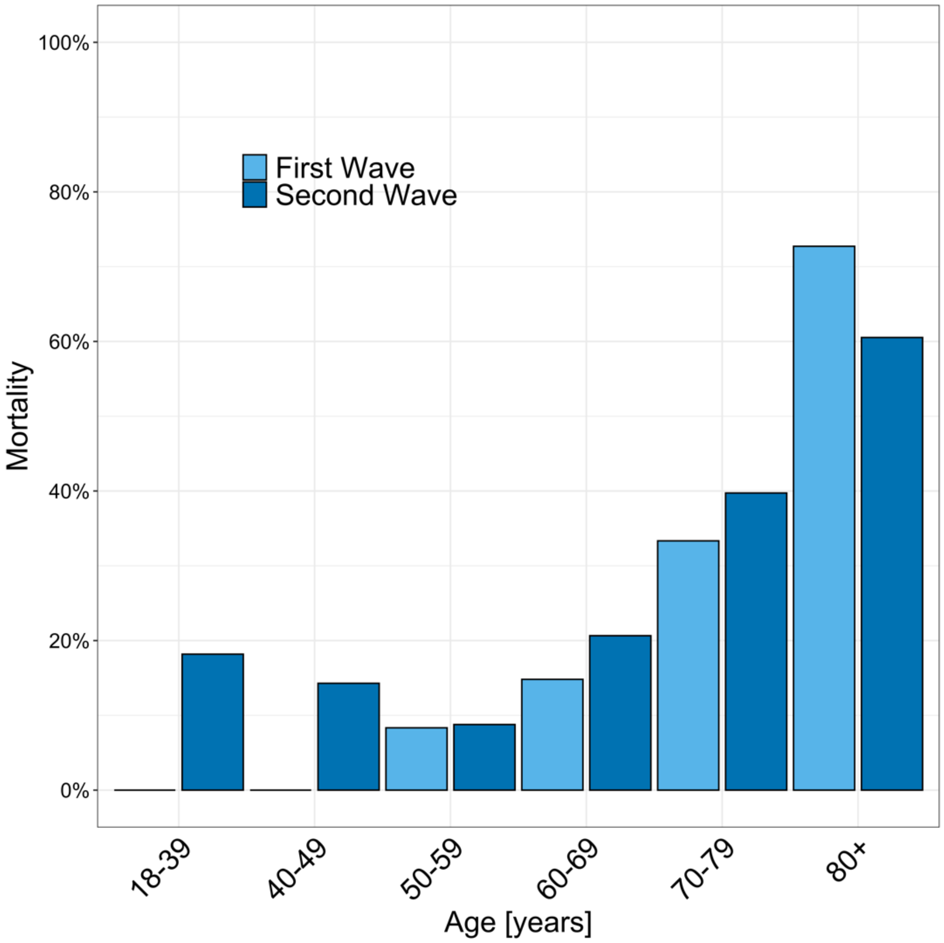

Supplemental Figure 1: Age and Hospital Mortality: hospital mortality in percent for different age groups in the first and second wave
